# Step type is associated with loading and ankle motion in tap dance

**DOI:** 10.1371/journal.pone.0303070

**Published:** 2024-05-29

**Authors:** Breanna A. Polascik, Yue Jiang, Daniel Schmitt

**Affiliations:** 1 Duke University Medical Center, Durham, NC, United States of America; 2 Department of Statistical Science, Duke University, Durham, NC, United States of America; 3 Department of Evolutionary Anthropology, Duke University, Durham, NC, United States of America; The Wingate College of Physical Education and Sports Sciences at the Wingate Institute, IL, ISRAEL

## Abstract

Tap dance generates forces and joint motions that can lead to injury; however, little is known about the magnitude of load across different tap steps. The purpose of this study was to calculate peak vertical forces, average vertical foot velocities, and maximum/minimum ankle angles produced by tap dancers with different levels of experience performing the toe cannon, heel cannon, flap, and cramp roll. This prospective cross-sectional study included 14 female tap dancers aged ≥18 years with varying tap experience. Participants were recorded by three cameras while performing a choreographed tap combination containing four steps of interest on a force platform. Adjusting for experience and dancer-level clustering, we identified the steps—cramp roll and toe cannon—that had the highest peak vertical ground reaction force, angles, and velocities compared to flap and heel cannon. There was no effect of experience. The results supported our hypothesis and provide new insights into step production. Over time, the larger forces associated with these steps could pose an increased risk of injury to bones and joints when compared to smaller forces, which may suggest the importance of adjusting routines to reduce or avoid injury.

## Introduction

Tap dance is a common recreational and professional art form that is characterized by lower extremity percussion. Integral to this lower extremity percussion are kinetic (ground reaction forces) and kinematic (limb velocities and joint angles) variables that influence the loads that the body experiences. Specifically, tap dancing generates ground reaction forces from loads applied by the body to the ground that can be injurious to bones and joints [1–3]. The peak vertical foot velocity (rate of descent of the foot) and ankle angles produced may vary by tap step and can have a strong effect on the ground reaction forces produced and resulting loads that the body experiences [4]. As a result of these recurring loads, tap dancers, like other dancers, are susceptible to potentially career-ending musculoskeletal injuries [5–14]. These injuries may be acute or chronic due to accumulated micro-trauma resulting from incorrect technique, lack of training or experience, and/or repetitive loads [3, 5, 7, 12, 14]. In fact, when compared to classical ballet (44.4%), jazz/contemporary (37.0%), and street dancers (41.2%), tap/folk dancers had the highest proportion (64.7%) of injuries ascribed to overuse [5].

Little is currently known about the biomechanics of tap dance. Mayers et al. (2010) found that in a group of professional tap dancers, the mean vertical ground reaction force was 2.06 ± 0.55% body weight (%BW) for each step studied (flaps, cramp rolls, pullbacks, and a self-selected “challenging” sequence) [15]. They speculated that force levels vary with experience but highlighted the need for further study. Rocha et al. (2017) explored joint range of motion in experienced tap dancers performing nerve beats, brush stamps, and heel ball walk tap steps and found that the knee and ankle had greater amplitudes of movement than the hip in all three steps [16]. While these studies form a solid foundation for understanding the link between tap dance, experience, and biomechanical variables that may increase forces leading to injury, much remains to be explored. For example, individual tap steps may produce distinct ranges of motion at different joints as well as vertical foot velocities (vertical descent measured from an ankle marker in the sagittal plane), influencing the ground reaction forces generated and thus potential for injury over time, but to our knowledge this has not yet been studied extensively in the literature.

Therefore, we aimed to calculate peak vertical forces, average vertical foot velocities, and maximum/minimum ankle angles produced by tap dancers with different levels of experience performing the toe cannon, heel cannon, flap, and cramp roll within a choreographed combination. We hypothesized (H1) that foot velocity and foot position would affect foot loading during tap dancing. Based on that, we predicted (P1) that cramp rolls and toe cannons because of their foot positions and rapidity would produce the greatest peak vertical forces, average vertical foot velocities, and maximum and minimum ankle angles compared to other step types. We also hypothesized (H2) that experience would affect foot loading. We predicted (P2) that peak vertical ground reaction forces will decrease with experience.

## Methods

### Participants

Fourteen tap dancers volunteered. Subject demographics were as follows (age: mean 20 ± 1.6 years, range 8–24 years; gender: 100% female; weight: 64.8 ± 15.2 kg, height 1.6 ± 0.1 m). Dancers were recruited between August 1, 2017 and January 30, 2018, from emails, fliers, and phone calls to dance studios, universities/colleges, and professional dance groups across the state of [removed for blind review]. Those who were <18 years old, had <6 mo of experience, had not danced within the last 4 years, or who had sustained musculoskeletal injuries or other injuries that caused participants to refrain from dancing (information collected through participant self-report) within the last 6 months were excluded. There was no restriction on gender, however, no males volunteered for the study. This study was approved by the Investigational Review Board for Human Research (E0130) at [removed for blind review]. Written informed consent was obtained from all participants.

### Measures

**Fig 1:**
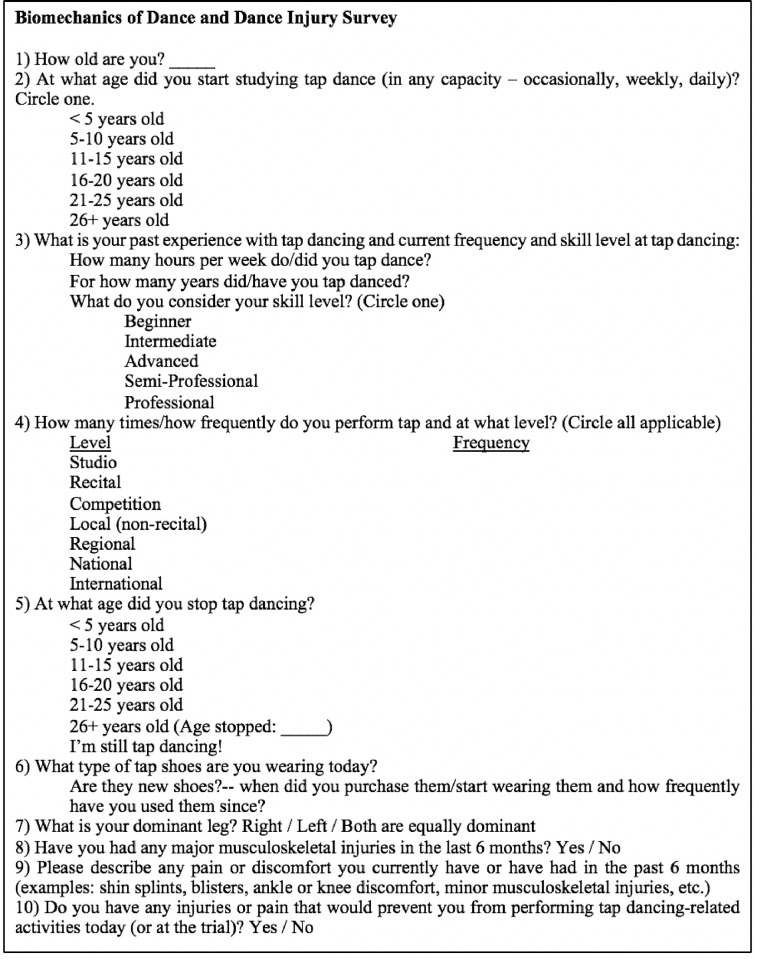
Survey assessing tap dance experience and presence or absence of current injuries.

Two questionnaires were developed and used in this study. The first was a written 10-question pre-study survey that assessed participants’ tap dance experience (years), tap dance history, and the presence or absence of current musculoskeletal injuries (Fig 1). The second was a post-study survey consisting of two questions (1. Did any of the tap steps in the combination hurt when you performed them? Please be specific. 2. Did you feel uncomfortable dancing with the equipment during the trials? Please explain.) that assessed subjects’ level of discomfort, if any, during the study.

### Design and procedures

Four tap steps were studied: toe cannon, cramp roll, flap, and heel cannon (Table 1). These are all common dance steps that were selected to be easy for any dancer to learn and to isolate different parts of the heel and toe: the cramp roll uses the ball and heel, the flap isolates the brush forward and slapping movement, the toe cannon incorporates the front edge of the toe, and the heel cannon incorporates the back edge of the heel.

**Table 1:** The four tap steps studied; Videos of each step: https://drive.google.com/drive/folders/1xZlw18eb71hMH0fJLD4QLSiaoRw0UCGh?usp=sharing.

Step	Definition
Toe cannon	jumping then landing the tip of the right toe, tip of the left toe, ball of the right foot, then ball of the left foot
Cramp roll	landing the ball of the right foot, ball of the left foot, heel of the right foot, heel of the left foot in quick succession to produce a four-sound step like a horse gallop or drum roll
Flap	brush forward with the right ball of the foot followed by stepping on the right ball of the foot
Heel cannon	four digs of the heel in quick succession (right, left, right, left) followed by a step on the right foot, where a dig is hitting the back edge of the heel to the ground, as if trying to dig a shovel in the ground

Two different tap combinations of equal duration and difficulty were choreographed in advance by a tap dancer with over a decade of experience ([removed for blind review]). Each of the two combinations contained the four tap steps to be evaluated (performed on the right side), interspersed with common beginner tap steps that were not being studied. The goal of this integration was to mirror a realistic setting in which tap steps are performed within a choreographed sequence. Subjects were not told which steps within the combination were being studied and thus were not aware that these four steps were specifically being evaluated. Each subject was randomly assigned to one of the two combinations and was given a pre-recorded instructional video made by the researcher ([removed for blind review]) to learn and practice their assigned combination one week before arriving to their scheduled study session.

The study was conducted at [removed for blind review]. Subjects wore tight-fitting, dark clothing and their personal tap shoes. Reflective motion capture markers (Bausch & Lomb Engineering, Santa Ana, California, USA) were placed in six locations on the right side of the body: lateral 5^th^ metatarsal head, lateral malleolus, lateral femoral condyle, lateral iliac crest, and greater trochanter. Subjects performed their designated combination on two Advanced Medical Technology, Inc. (AMTI) force plates (Sampling frequency 1200 Hz; OR6 -7-OP-1000; 508 cm x 464 cm x 82.5 cm each; Advanced Mechanical Technology, Inc., Watertown, Massachusetts, USA) which output force (Newtons) in three orthogonal directions. The two force plates were positioned as close together as possible without touching, so the subject could comfortably keep one foot on each plate. Subjects were allowed to warm up in their normal manner and practice the tap combination on the force plates to become familiar with the setting. If the subject felt uncomfortable or expressed any concern about the surface, they were allowed to leave the study.

Once the subject was ready, three Go Pro Hero Blacks (120 Hz; Go Pro, Inc., San Mateo, CA, USA) positioned on the ventral and lateral sides of the subject recorded all activity. Two cameras (ventral and lateral) recorded the full body, while the third (lateral) was zoomed in on the feet. The cameras were calibrated using a static calibration star (Peak Performance Technologies, Inc., Englewood, CO, USA). Each trial began with three right-foot stomps by the subject on the right force plate. The stomps were visible on the video and recorded by the force plate, allowing precise synchronization of video and force data. The subject performed three trials, with each trial consisting of the subject performing their designated combination one time through at a self-determined relaxed pace. The goals of using a relaxed pace were twofold: to gain an understanding of the forces generated in even the more casual tap dance scenarios and to allow tap dancers of all levels of experience to successfully perform the combination.

The force plates recorded ground reaction forces for the four selected tap steps. The video data were used for positioning (x, y position of the body and body segments), spatiotemporal (timing), and kinematic (angles and velocities) data. Force data was transferred to MATLAB (The MathWorks, Inc., Natick, Massachusetts, USA) using purpose-written code to find the peak values for the ground reaction force for each impact of the foot during each of the four steps (Newtons, normalized to weight). The peak values for each step type were then averaged between the 3 trials for each subject. Kinematic data from the video cameras were transferred to DLT dataviewer within MATLAB [17], and x, y coordinates were digitized for the metatarsal, ankle, knee, and hip on the right side. Maximum and minimum ankle angle (degrees) and average velocity (m/s) of the foot were calculated during the four tap steps using MATLAB. Specifically, ankle angle was calculated by measuring the angles created between the 5^th^ metatarsal head, lateral malleolus, and lateral femoral condyle markers during the tap step in question. Velocity was calculated by averaging the rates of vertical descent of an ankle marker from the start of the movement (beginning of vertical descent) to the generation of the ground reaction force for each force-generating movement within a tap step.

### Statistical analysis

We observed three replicates for each step type for our fourteen participants, resulting in 42 observations for each step type. For each of the five continuous outcomes of interest (vertical peak force, average velocity, minimum ankle angle, maximum ankle angle, and range of motion), we fit a linear model with the step type as the main predictor of interest, additionally adjusting for years of experience. Liang-Zeger (clustered) standard errors were used to account for correlation induced by within-participant clustering [18]; in the case of non-constant residual variance, a sensitivity analysis using Huber-White (heteroskedasticity-robust) standard errors was conducted to evaluate robustness of conclusions to assumption violations [19]. Step-down conditional contrasts were estimated to examine pairwise comparisons adjusting for experience and were adjusted for multiplicity using the Bonferroni-Holm method [20]. A linear mixed model with random intercepts by participant was considered as a modeling approach but was ultimately decided against because of concerns regarding model assumptions for the hierarchical structure not being met. All analyses were conducted at a significance level of 0.05 using R version 4.1.1 [21].

## Results

### Demographics and survey data

Half of the dancers (n = 7) started dancing when they were 5 years old or younger, while others started at 5–10 years old (n = 5), 11–15 years old (n = 1), and 16–20 years old (n = 1). All but two dancers were still tap dancing at the time of the study. These two dancers stopped dancing because of other commitments, not because of injury, and were included as they had danced within the past 4 years. Experience level was determined by years of tap dance experience, which ranged from 3 to 19 years (mean 14 years; *SD* 4.7 years).

### Forces, velocities, and ankle angles

Table 2 demonstrates the peak vertical forces, average vertical velocities and minimum and maximum ankle angles by step type. In Table 3, we display pair-wise differences in each of the four measurements between the six combinations of steps, adjusted for years of experience (e.g., “C-F” indicates the difference between cramp roll and flap).

**Table 2:** Peak vertical forces, average vertical velocities, and minimum and maximum ankle angles, by step type.

	Toe Cannon	Cramp Roll	Flap	Heel Cannon
	Mean (*SD*),	Mean (*SD*),	Mean (*SD*),	Mean (*SD*),
	Min.–Max.	Min.–Max.	Min.–Max.	Min.–Max.
Peak Vertical Force (% body weight)	1.10 (0.59),	1.12 (0.44),	0.79 (0.20),	0.81 (0.27),
	0.15–4.04	0.28–2.48	0.55–1.36	0.47–1.63
Average Vertical Velocity (m/s)	0.81 (0.58),	0.56 (0.40),	0.32 (0.20),	0.42 (0.34),
	0.20–3.08	0.15–1.85	0.09–0.89	0.11–2.04
Min. Ankle Angle (Degrees)	95.0 (9.58),	92.1 (8.30),	95.6 (9.27),	93.4 (7.16),
	71.9–112.2	74.7–106.47	75.5–114.3	79.1–107.8
Max. Ankle Angle (Degrees)	155.0 (11.0),	133.9 (9.59),	130.8 (10.6),	111.4 (6.60),
	135.3–178.6	116.3–151.2	101.6–146.9	96.6–123.9

**Table 3:** Pairwise differences in peak vertical forces, average vertical velocities, and minimum and maximum ankle angles, adjusted for years of experience.

Difference* (SE), p-value	Peak Vertical Force	Avg. Vertical Velocity	Min. Ankle Angle	Max. Ankle Angle
C–F	**0.332 (0.103)**	**0.239 (0.060)**	-3.508 (1.290)	3.146 (3.434)
	**p = 0.007**	**p = 0.002**	p = 0.018	p = 0.380
C–H	**0.313 (0.084)**	0.141 (0.046)	-1.312 (1.274)	**22.54 (2.488)**
	**p = 0.002**	p = 0.009	p = 0.322	**p < 0.001**
C–T	-0.028 (0.123)	**-0.248 (0.060)**	-2.931 (1.381)	**-21.08 (2.379)**
	p = 0.833	**p = 0.001**	p = 0.054	**p < 0.001**
F–H	-0.018 (0.045)	-0.098 (0.048)	2.196 (1.070)	**19.40 (2.358)**
	p = 0.692	p = 0.063	p = 0.061	**p < 0.001**
F–T	**-0.359 (0.104)**	**-0.487 (0.093)**	0.577 (1.729)	**-24.22 (3.871)**
	**p = 0.004**	**p < 0.001**	p = 0.744	**p < 0.001**
H–T	**-0.341 (0.094)**	**-0.389 (0.062)**	-1.619 (1.932)	**-43.62 (3.036)**
	**p = 0.003**	**p < 0.001**	p = 0.417	**p < 0.001**

*Adjusted for years of experience; **Bold** indicates statistically significant adjusted difference at Bonferroni-Holm-corrected 0.05 significance level. C: Cramp Roll; F: Flap; H: Heel Cannon; T: Toe Cannon

Models for peak vertical force and average vertical velocity displayed evidence of non-constant variance; however, in using Huber-White standard errors as a sensitivity analysis, neither coefficient nor standard error estimates changed appreciably, and all conclusions remained the same. Flaps and heel cannons tended to have lower peak vertical force, average vertical velocity, and maximum ankle angle compared to toe cannons and cramp rolls. In examining pairwise comparisons adjusted for years of experience and dancer-level clustering, toe cannon–flap, toe cannon–heel cannon, and cramp roll–flap differences in both peak vertical force and average vertical velocity were statistically significant, with toe cannons having lower peak vertical force and average vertical velocity compared to flaps or heel cannons, and cramp rolls having higher peak vertical force and average vertical velocity compared to flaps. We additionally identified statistically significantly higher peak vertical force in cramp rolls compared to heel cannons, and statistically significantly lower average vertical velocity in cramp rolls compared to toe cannons.

Minimum ankle angles were similar across all four steps, with no statistically significant pairwise differences identified, but all differences apart from cramp roll–flap were statistically significant regarding maximum ankle angle. Range of motion tended to be lower in flaps and heel cannons compared to cramp rolls and toe cannons, with toe cannons having the highest range of motion among all four steps. This was primarily driven by differences in the maximum angle degree.

## Discussion

We aimed to calculate peak vertical forces, average vertical foot velocities, and maximum/minimum ankle angles produced by tap dancers with different levels of experience performing the toe cannon, heel cannon, flap, and cramp roll within a choreographed combination. Our results demonstrate that toe cannons and cramp rolls tended to have higher peak vertical forces, average vertical velocities, and maximum ankle angles when compared to flaps and heel cannons, with several statistically significant pairwise differences. Over time with repetition, the larger forces generated by toe cannons and cramp rolls could pose increased risk of overuse injury to bones and joints when compared to smaller forces [1, 3]. Furthermore, the execution of toe cannons and cramp rolls requires the ankle to be in a loose-packed joint position, which is unstable, possibly increasing the risk of acute mechanical injury or ligamentous/muscular injury from sprains, but further study is warranted.

Our results are consistent with Mayers et al. (2010), who looked at flaps, cramp rolls, pullbacks, and a dancer-selected sequence, and found that in a group of professional tap dancers, the mean peak ground reaction force was significantly higher in cramp rolls (2.39 BW) than in flaps (1.53 BW) [15]. One point of interest is that our cramp roll and flap peak vertical ground reaction forces were lower (1.1% BW) than those found by Mayers et al. (2010). This may be explained by many variables. The studies were conducted in different settings, with our use of two force plates vs one used by Mayers constituting a larger or more restrictive dance space. Mayers also had participants repeat each step 4–8 times in succession while our steps were integrated into a combination. This may have produced different weight shifts that altered the forces measured. The foot velocity was guided by a metronome in Mayers’ study while ours was at self-selected relaxed pace, which could have influenced the differences. Our results therefore may be an underestimate or a broader picture of what dancers are experiencing. The tap steps chosen in our study were all fairly simple and it is likely that forces, velocities, and angles may be different if looking at more advanced steps with greater emphasis on jumping or speed. However, the patterns of increased force are consistent. Future study to further elucidate these differences is warranted. Our study added to Mayers’ findings in a few ways. First, we studied two additional common tap steps—toe cannons and heel cannons—that, to our knowledge, have not yet had this analysis in the literature since little currently exists on the forces generated by different tap steps. Second, the subjects in our study were younger nonprofessional athletes with different levels of experience and training, whereas the subjects in Mayers et al. (2010) were all professional tap dancers, resulting in a different study population.

We also found that experience does not explain the variation in force, velocity, or ankle angle. To our knowledge, this is the first study in the tap literature to examine the impact of experience on these biomechanical variables. This does not entirely agree with the proposition by Mayers et al (2010), who found some effect of experience and with Rocha et al (2017) who found improved motor control in experienced dances. However, our data are consistent with, Klopp (2017) who found that Irish dancers’ experience level was not correlated with peak force generated during Irish dance landings [22]. This is clearly an area that deserves further study.

Biomechanics have been studied in other styles of dance including ballet, modern, Irish, Flamenco, and Bharatanatyam, among others [23–25]. Irish dancers, for example, experience ground reaction forces of up to 4.5 times body weight when performing the rock step [6, 26]. Similarly, Bharatanatyam dancers generate ground reaction forces of 4–5 times body weight when performing the tatta adavu step [27]. Our study demonstrated ground reaction forces of up to 1.1% body weight when performing the cramp roll and the toe cannon. Although these other dance styles focus on percussive rhythms of the lower extremity as does tap dance, variable methods, dance steps, and units of measurement limit the ability to make direct comparisons. For example, the tatta adavu step constitutes forceful slamming of the flat foot on the ground. This is a very different movement from any of the tap steps studied in this study, but may merit a more direct comparison to a flat foot tap stomp in a future study. However, this provides a general understanding of the variability in forces produced by different dance styles and further underscores the need for future research.

Our study had several limitations. First, while our study population was representative of tap dancers with a range of experience, recruitment of novice dancers proved challenging. Inclusion of even more beginners in future studies may be warranted. Second, since the setup was not a completely natural environment in which one would normally tap dance, it is possible that dancers may have slightly modified their movements. However, in the post-study survey, all dancers reported that they did not feel that their performance was inhibited by the setup or the equipment and had only minor comments about markers or tape, so the effect of this on the results was likely minimal. Third, we used years of tap dancing as a measure of experience because it is an objective foundation from which to begin investigation. Future studies may use a more nuanced definition of experience, such as subjective skill level as determined by a panel of professional tap dancers or a combination of years of experience, aesthetics, sound, and quality of training. Fourth, there was no data to specify the pace each dancer utilized for their “self-selected relaxed pace.” Although a self-selected relaxed pace was chosen to address specific concerns as mentioned in the methods section, this provides an opportunity for future studies to look at differences when performing tap steps at various predetermined speeds. In addition, we report ankle angles across steps but not in reference to each person’s neutral position, which was not recorded in a static stance. Finally, we chose to study simple tap steps with minimal weight transference because they represent common tap steps that most beginners would be able to perform easily with one foot on each force plate in a practice setting. We also focused our analysis on the right side of the body for consistency and to minimize variance as the vast majority of the participants were right side dominant. Future studies may build upon these findings by studying more complex tap steps with greater weight transference or left-right differences.

## Conclusions

In conclusion, this study builds on the existing literature to further explore the forces, velocities, and ankle angles produced in tap dance as well as their relationship to years of experience. The findings demonstrate that cramp rolls and toe cannons are overall higher impact steps in terms of forces, velocities, and maximum ankle angles generated than heel cannons and flaps and that the magnitude of peak vertical force is not correlated with experience. Knowledge gained from this study increases our understanding of the biomechanics of tap dance in tap dancers with varying levels of experience. Tap dancers and instructors of all experience levels could potentially utilize these findings to aid in the development of lower- or higher-impact choreography. Knowledge gained in this study may also supplement understanding of similar impacts generated during other types of human performance and facilitate future study about how these variables may influence risk of injury in tap dancers.

## Supporting information

S1 Data (XLSX)
